# Challenges of bacteriophages application in controlling bacterial plant diseases and how to overcome them

**DOI:** 10.1186/s43141-023-00549-y

**Published:** 2023-10-10

**Authors:** Esraa M. Halawa

**Affiliations:** Botany and Microbiology Department, Faculty of Science, Cairo University, Giza, 12613 Egypt

**Keywords:** Virus, Bacteriophage, Phage therapy, Biocontrol, Plant diseases, Challenges

## Abstract

Due to the emergence of antibiotic-resistant bacteria in agricultural sector, controlling bacterial plant diseases using antibiotics has become challenging. Researchers have turned to alternative methods, such as using bacteriophages as a biocide for plants instead of antibiotics, to control pathogenic bacterial plant diseases. However, the application of bacteriophages as a biocide in agriculture faces several challenges that may impede its success. In this review article, we discuss the various issues that could lead to the failure of its application. We also propose solutions to address each problem to increase awareness and familiarity before implementing the method to better ensure its success.

## Background

Viruses are microscopic, obligate intracellular parasites that replicate and propagate by exploiting the host cell machinery. Therefore, they depend entirely on their host cells for survival [1]. Viruses cannot survive for long periods outside their host cells, which means their life cycle is entirely dependent on their hosts [2]. Due to their tiny size and complete dependence on host cells, viruses can infect a wide range of organisms, including bacteria, plants, and mammals [3]. Bacteriophages, or phages, are the most abundant viruses on Earth. They infect bacteria and archaea [4], and the term bacteriophage is a combination of two words: "bacterio," derived from "bacteria," and "phage," derived from the Greek "phagein," meaning "to devour" [5]. Since phages display high specificity for infecting and destroying bacteria, they are present wherever bacteria are found and have been utilized to eliminate pathogenic bacteria [6]. Bacteriophages are generally classified into two categories: virulent phages and temperate phages [7]. In recent years, there has been growing interest in using bacteriophages to control plant diseases caused by bacterial pathogens. Several studies have demonstrated the effectiveness of bacteriophages in controlling plant diseases. For example, a study conducted by Abuladze et al. (2008) showed that applying bacteriophages reduced the severity of bacterial spot disease in tomato plants [8]. Despite the potential benefits of using bacteriophages for plant disease control, some challenges still need to be addressed. This review article aims to summarize the challenges that may hinder successful phage applications and provide solutions to overcome these challenges.

### The mechanism of interaction between phage and bacteria (Fig. 1):

**Fig. 1:**
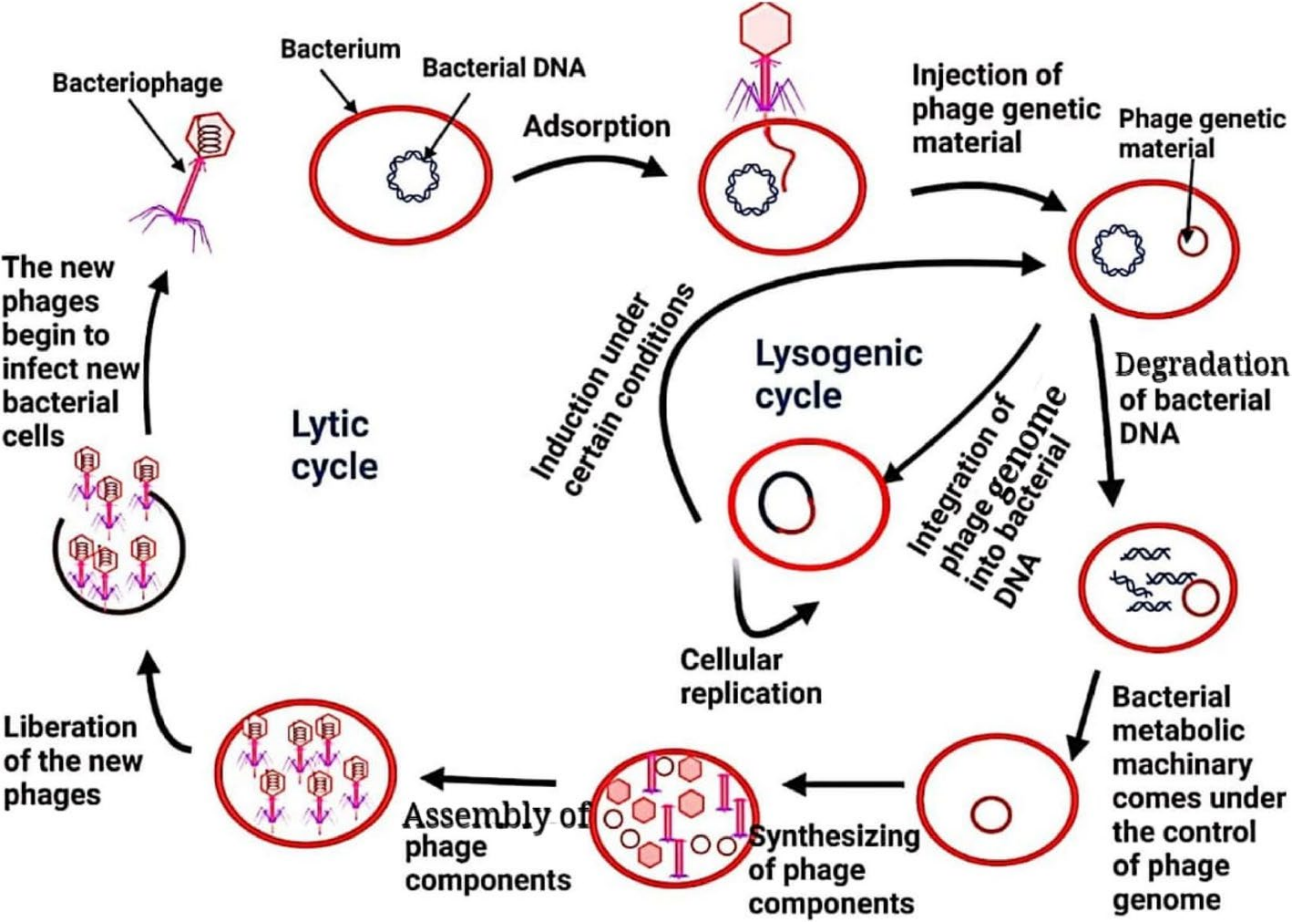
Mechanism of interaction between bacteriophage and bacteria. "Figure created with http://biorender.com"

A - **The first step of infection is: Adsorption of phage to the bacterium surface**

Infection of bacterial cells by virulent or temperate phages begins with the phage adsorption organelle binding to bacterial-specific receptors on the bacterial surface. The bacterial and viral attachment sites are different. Examples of cell surface components that can serve as phage receptors include proteins, lipopolysaccharides in gram-negative bacteria, and peptidoglycan and teichoic acid in gram-positive bacteria. It is not uncommon for two or more phages to detect the same receptor site on the same bacterium; a small number of phages may even be capable of binding to two or more different receptors [9].

In addition to the secondary function that a phage utilizes, the receptor site on the bacterial surface performs primary functions. Among these primary functions is the transport of nutrients (such as vitamins, sugars, and amino acids) by numerous proteinaceous receptor sites and other receptors associated with organelles of specific functions, such as flagella and conjugative pili [10].

Initially, the phage adsorption organelle binds reversibly to the bacterial receptors. The bacteriophage can be separated while retaining its infectious properties, such as through diluting the reaction mixture or bactericidal killing. The process that leads to the penetration of the phage's genetic material into the bacterial cell ultimately renders the relationship irreversible [11].

B - **The second step of infection: Injection of phage genetic material into the bacterial cells**

After attaching the phage adsorption organelle to the bacterial receptor, the phage begins inserting its genetic material into the cytoplasm. This occurs due to the tail sheath's contraction, which functions as a hypodermic needle to inject the phage genome into the cell membrane and wall. Only the genetic material of the phage enters the bacteria; the rest remains outside [12].

C - **The third step of infection**

This step differs in virulent phages from temperate phages. Therefore, we discuss this step in both types of phages separately as follows:

- **The third step of infection in virulent phage** 

After the phage genetic material enters the bacterial cytoplasm, the phage synthesizes virus-encoded endonucleases to degrade the bacterial chromosome [13]. Subsequently, the phage genome takes over the bacterium's metabolic machinery, converting it into a "factory" for manufacturing phage components, including capsomeres, sheath, base plates, tail fibers, and phage enzymes. Once the phage components' synthesis is complete, they assemble to form new phages called progeny phages [9]. Phage proteins like holin or lysozyme then disrupt the bacterial cell wall to release the new phages, which ultimately infect new cells [14, 15].

- **The third step of infection in temperate phage**

After entering the bacterial cytoplasm, the genetic material of a phage integrates into the bacterial chromosome and becomes a prophage, which makes the bacterium a lysogen. Lysogeny is the process by which a temperate phage infects a bacterium. During reproduction, as the bacterium replicates its chromosome, it also replicates the phage's DNA and transfers it to new daughter cells [16]. The presence of a phage genome in the bacterial chromosome can alter the bacterium's phenotype by introducing additional genes, such as toxin genes, which can increase bacterial virulence. This alteration in the host phenotype is called "lysogenic conversion" or "phage conversion".

In certain bacteria, such as *Vibrio cholera* and *Clostridium botulinum*, the absence of the prophage results in decreased virulence. The phages that infect these bacteria carry toxin genes in their genomes which, when expressed, increase the host's pathogenicity. Phage-encoded toxins can induce paralysis in *Clostridium botulinum* and severe diarrhea in *Vibrio cholera* [16–19].

During lysogeny, the prophage persists in the bacterial chromosome and replicates as a unit without destroying the bacterial cell. However, under certain conditions, lysogenic phages can be induced to follow a lytic cycle and undergo lysogeny in a newly infected cell [16].

-** Application of viruses in phage therapy**

### The use of phages in the treatment of bacterial diseases

Phages are becoming increasingly popular as alternative biocontrol agents for controlling microbial resistance [20]. Phage therapy is the use of phages to treat infections caused by pathogenic bacteria [21]. Phages are highly effective in antimicrobial phage therapy because they have the ability to specifically identify, bind to, multiply within, and lyse bacterial cells. Bacteriophages can effectively target both gram-positive and gram-negative bacteria that are resistant to antibiotics, and they typically act with a high degree of specificity [22].

### There are several potential advantages to using phages in disease control

Phages offer numerous benefits, including their ubiquitous presence wherever bacteria exist and their ability to replicate as long as bacteria are present. Additionally, phages possess high specificity to infect target bacteria without harming others, are non-toxic to eukaryotic cells, can be easily and inexpensively prepared and produced, and can be stored for months under preservation conditions without significant loss in titer [23]. Phages are an environmentally friendly biocide that can eliminate antibiotic-resistant bacteria [24], and can also be used to produce crops free of chemical pesticides, which are increasingly in demand among consumers [24, 25].

### Challenges that may hinder successful phages application for plant disease control and how these challenges can be overcome (Table 1):

**Table 1:** Challenges that may hinder successful phages application and how they can be overcome

Challenges that may hinder successful phages application	How they can be overcome
1) Inactivation of phages by UV irradiation	- Using a protective formulation that protects phages against UV - Applying phages after sunset
2) Poor persistence of phages in the rhizosphere and phyllosphere	- Accompanying phages by a viable host - Avoiding daylight during phages application
3) Possibility of lysogens or pseudo lysogen production	- Using only lytic phages
4) The difficulty of eliminating every member of a particular bacterial genus or species due to the narrow host range for phages	- Development of phage cocktails
5) Instability of phages survival under improper storage conditions	- Keeping phages refrigerated and protected from light - Production of stable bio formulation and conversion of phage formulation from liquid to powder state
6) Possible development of phage resistance in the bacterial host	- Development of phage cocktails
7) The inability of phages to disperse or interact with their target bacteria when there is a lack of moisture on the leaf surface	- Applying phages when free moisture is expected to stay on the leaves (such as dew or rain)
8) The difficulty of applying phages evenly over large tree leaves	- Applying phages via tree vascular system

1. **Inactivation of phages by UV irradiation from the sun**

UV radiation can damage the DNA of phages, potentially inhibiting DNA replication. To mitigate this issue, phages can be applied after sunset to reduce the damaging effects of UV [26]. Research has demonstrated that the application of phages onto tomato leaves during the evening hours leads to an extended duration of phage persistence within the phyllosphere. This prolonged persistence provides phages with increased opportunity to infect and eliminate their bacterial targets [26].

Alternatively, a formulation that shields the phages from UV radiation can be used. Born et al. (2015) examined a range of substances to determine their effectiveness in providing UV protection for phages. They found that natural extracts from carrot, red pepper, beetroot, casein, soy peptone, purified aromatic amino acids, astaxanthin, and tween 80 all provided UV protection, without inhibiting phage infection or stability [27]. Therefore, various compounds may enhance phage performance in the phyllosphere, with the essential criterion being their ability to absorb UV to reduce phage exposure. It has also been demonstrated that biodegradable polymers [28], Congo red dye [29], iron oxide particles in groundwater [30], ferric chloride coagulant, and humic acid [31] all have UV protection properties for phages.

According to Templeton et al. (2005), certain organic colloidal particles have the ability to protect phages from UV light, whereas inorganic kaolin clay particles do not offer such protection [31]. In a subsequent study by Templeton et al. (2006), it was observed that the UV inactivation of both phages in "raw" groundwater was significantly lower than in EDTA-preserved groundwater. This was attributed to the association of the phages with UV-absorbing iron precipitate particles. A phage elution technique confirmed that a considerable proportion of the phages that survived UV exposure were associated with particles [30].

Khalil et al. (2016) conducted a study which demonstrated that incorporating Poly-γ-glutamic acid (γ-PGA), a biodegradable polymer, into phage formulations yielded protection against UV damage, high temperatures, and extreme pH values. Additionally, these biodegradable polymers prolonged the persistence and viability of phages, thereby enhancing the efficacy of biocontrol using phages in comparison to non-formulated phages. The authors suggest that this protection may be attributed to physical shielding of the virus particle by γ-PGA, which reduces the levels of heat reaching the viral particles. Alternatively, the high amino acid content of γ-PGA may promote virion survival [28].

According to a recent investigation carried out by Wdowiak et al. (2023), it was revealed that Congo red, a dye commonly utilized in laboratory research, exhibits exceptional protective characteristics towards non-enveloped phages against extended exposure to UV radiation. In contrast, non-protected phages without Congo red were completely deactivated within one minute of UV irradiation. The dye functioned as a "molecular sunscreen," shielding phages from the damaging effects of UV radiation. This outcome presents a promising solution for overcoming the issue of phage inactivation by UV radiation [29].

(2) **Poor persistence or short-lived phages in the rhizosphere and phyllosphere**

The region surrounding the roots of a plant is known as the rhizosphere [32]. The aerial parts of a plant above the ground are known as the phyllosphere [33]. Phages are poorly persistent in the rhizosphere due to several factors, including low rates of phage diffusion through the heterogeneous soil matrix, the ability of phages to become trapped in biofilms and reversibly adsorb to soil particles like clay, low soil pH, which can also render phages inactive, and physical refuges that prevent bacteria from contacting phages. Notably, a few phages survive due to low phage diffusion rates and high phage inactivation rates. The poor persistence of phages in the phyllosphere is attributable to environmental factors such as temperature, desiccation, and exposure to specific chemical pesticides such as copper bactericides on leaf surfaces [34].

These issues can be resolved if viable hosts and phages are present (propagating bacterium strains). A viable host can be an avirulent strain of the pathogenic bacterium being targeted or another naturally occurring bacterial strain in the environment. These bacteria may increase the likelihood of continued phage propagation to counteract expected losses from environmental factors such as sunlight and dehydration. Since they readily establish themselves in the phyllosphere and can compete with pathogenic bacteria, nonpathogenic epiphytes may be the best candidates for this role. In addition, nonpathogenic or attenuated phytopathogen strains, which no longer cause disease, can also be used for phage propagation. Since these bacterial strains are the same species as the bacterial pathogen, they will likely propagate most phages and exert some antagonistic effects [35]. According to Nagai et al. (2017), a study was conducted on broccoli plants infected with black rot disease to investigate the potential of using bacteriophages accompanied by avirulent bacterial species as a means of enhancing phage persistence on the leaf surface and improving the biocontrol of the disease. The results of the study indicated that this approach was successful in achieving these objectives [36]. It has also been reported that applying phages in combination with avirulent bacterial strains has proven to be effective in the biocontrol of tobacco wilt disease [37].

Additionally, avoiding direct sunlight during application has been observed to improve phage-based biocontrol. Delivering phages into tomato leaves in the evening causes them to remain longer in the phyllosphere, giving them more opportunities to infect and destroy their bacterial hosts [38].

(3) **Possibility of lysogen or psuedolysogen production**

The persistence of the phage genome within a host cell can provide superinfection immunity, thereby reducing the biological efficacy of the phage and conferring additional characteristics on the target bacterium. For example, the phage RSS1, which is present in *Ralstonia solanacearum* in a persistent infective state, enhances the virulence of the bacterial host on tomato [39]. To avoid issues related to lysogeny, it is recommended to use only lytic (virulent) phages for biocontrol [24, 40]. According to the findings of Álvarez et al. (2019), three lytic phages were isolated from river water and found to exhibit activity against *R. solanacearum*, resulting in significant biocontrol efficacy against bacterial wilt disease. This study represents the first reported instance of successful biocontrol of *R. solanacearum* using single or combined bacteriophages delivered through irrigation water under conditions that mimic those of natural settings [41]. Thepa Magar et al. (2022) have conducted a recent study that examines the biocontrol potential of two isolated lytic phages against the invasion of *Ralstonia pseudosolanacearum*, the causative agent of bacterial wilt disease in tomato plants. The study found that treatment with either of the two phages alone or in combination resulted in a noteworthy decrease in the incidence of bacterial wilt [42]. There are also several studies that have utilized lytic phages and demonstrated their efficacy as a biocontrol agent against various plant diseases [43–48].

(4) **Narrow bacterial host range for phages**

Phages could demonstrate narrow host ranges as they are highly host-species-specific and commonly only can infect one bacterial species or even a subspecies [49]. The production of phage cocktails can resolve this issue [24]. The utilization of phage cocktails has demonstrated a broad spectrum of host range and effectiveness in the biocontrol of various bacterial plant diseases [50]. Iriate et al. (2012) conducted a study which demonstrated the efficacy of phage cocktail application in the biocontrol of *Xanthomonas perforans*, a causative agent of disease in tomato plants [51]. Similarly, Wang et al. (2019) conducted a separate study which confirmed the efficacy of phage cocktails in the biocontrol of tomato wilt disease [52]. According to a study carried out by Wei et al. (2017), the utilization of phage cocktails has proven effective in controlling potato bacterial wilt disease [53]. Also, there have been numerous studies that have applied phage cocktails and reported successful biocontrol outcomes for various plant diseases [50, 54–58].

Cocktail phages may be effective against different strains of the same bacterial species. However, based on their lytic activities, their combined efficacy in killing target bacteria might exceed expectation. This phenomenon, which may be beneficial and valuable therapeutically, is known as synergy [59, 60]. According to Schmerer et al. (2014), such synergy can be achieved when one phage facilitates infecting the same bacterium for another phage. They isolated phages from sewage and observed that these phages caused a mucoid *E. coli* strain to produce numerous plaques. The combined activity of two phages, J8-65 (producing turbid plaques with a halo effect) and T7 (forming small plaques), increased the host bacterial killing effectiveness by 10–100 fold compared to each phage acting alone [60]. Understanding the possibility of achieving synergy can significantly improve the production of phage preparations for phage therapy, as it increases their potential efficacy [22].

(5) **Instability of phage survival under improper storage conditions**

The successful use of phages depends on their stability. Temperature is the most significant factor affecting phage stability since it affects phage proliferation, action, and preservation [61]. Other factors that affect phage stability include techniques used to make phage compositions, substances and components they contain, forms in which they are used, preservation conditions kept, and application methods [62]. The issue of phage instability under improper storage conditions can be resolved by keeping phages cold and protected from light. In this manner, phage cocktails can be stored for months without significant titer loss. Also, depending on bacteriophage type, they may be frozen with or without propagating bacterium [35].

An alternative approach for addressing the issue of phage instability due to inadequate storage conditions involves the production of a stable bioformulation, accompanied by the transformation of the phage formulation from a liquid to a powdered state. This method of preparation enables the phage formulations to withstand harsh environmental conditions for an extended duration [63]. A practical investigation was carried out by Leung et al. (2018) to evaluate the stability of bacteriophages in powder form during storage at ambient temperature. According to the findings of this study, spray dried bacteriophage powder can be successfully stored for up to one year with vacuum packaging at 4 °C and 20 °C [64]. Similarly, Chang et al. (2019) conducted a study to examine the storage stability of phage powder under normal environmental conditions. As per the results of this study, dried phage powders are physically and biologically stable during long-term storage at surrounding temperature [65].

(6) **Possible development of phage resistance in the bacterial host**

Like with antibiotics, bacteria can develop resistance to phages through various mechanisms [39]. These defense mechanisms include changes in the bacterial surface receptors, such as CRISPR/Cas [66], mucous production [67], DISARM [68], lysogen production [69, 70], BREX [71], RM [72], and nine other novel systems [73]. This issue can be resolved through the production of phage cocktails that combine phages with narrow, wide, and/or host range mutant combinations [39, 40].

The utilization of phage cocktails has demonstrated a decrease in bacterial resistance development to bacteriophages, as well as their efficacy in controlling bacterial plant diseases [74, 75]. In a study conducted by Kim et al. (2022), it was reported that phage cocktails successfully inhibited the emergence of *P. carotovorum* strains resistant to phages. Additionally, the phage cocktails exhibited biocontrol properties against soft rot disease in napa cabbage [76].

In order to prevent and counteract the emergence of microbial resistance, several supplementary strategies have been proposed. These include the utilization of diverse therapeutic approaches in combination, the implementation of mutant phages derived from the wild type bacteriophage to regain activity against bacteria [77], and the isolation of novel or modified phages [78] that exhibit effectiveness against resistant microorganisms. Sieiro et al. (2020) have suggested the implementation of phage cocktails as a biocontrol agent in agriculture, in combination with the use of endolysins and antibiotics as part of an integrated approach to manage microbial infections and impede the emergence of resistant bacterial strains [79].

(7) **Inability of phages to disperse or interact with their target bacterium when there is a lack of moisture on the leaf surface **[80].

To ensure longer exposure time with the target bacteria, the issue can be resolved by applying phages during periods of extended free moisture on the leaves, such as during rainfall or when dew is present on the leaves at night and in the early morning [80].

(8) **Potential difficulty in applying phages evenly over large tree leaves**

Phages applied through the vascular system of the tree can resolve this issue. In addition, this type of phage application may facilitate the systemic delivery of the phages throughout the tree's vascular system and water flow [81].

### Some additional applications that can exploit viruses in their applications

The use of phages in the treatment of diseases, as mentioned earlier, is one of the applications of viruses. However, viruses can also be employed in the following applications:

2 - **Application of viruses in Nanotechnology**

Viruses are excellent bio-nanomaterial due to having unique characteristics. Viruses are minuscule in size, modifiable, able to carry materials, and have high self-assembly precision. These distinctive properties contribute to their application in nanoscience and nanotechnology [82]. Indeed, many different viruses have been successfully applied in nanotechnology. For example, mammalian viruses have been applied in the medical field as vectors. Plant viruses can be produced easily and safely as they do not infect human or animal cells. In addition, plant viruses possess a symmetrical structure and biodegradability. Recently, nanomaterials have been used to transport active molecules and drugs into cancer cells using plant viruses. Therefore, plant viruses offer a novel and potent therapy against cancer. CPMV, TMV, and PVX are the viruses from which the most well-known viral vectors are generated [82–85].

3 - **Application of viruses in vaccine delivery**

Viruses such as adenovirus can be used as vectors to deliver vaccine antigens to target cells. Adenovirus is characterized by its ability to infect a wide range of hosts, induce high levels of transgene expression without integrating its genes into the host genome, and induce the host's innate immune responses via toll-like receptor-independent and toll-like receptor-dependent pathways. All these distinctive features of adenovirus have led to its utilization in many vaccine developments, including HIV vaccines [86, 87]. Another example of a virus exploited as a vaccine vector is the alphavirus. Alphaviral vaccine vectors have been explored in myriad applications for cancers, HIV, and the human parainfluenza virus. Alphavirus can create a proper environment for cross-priming vaccine antigens by inducing apoptosis in some cells [88–90].

4 -** Application of viruses as a bioinsecticide**

As a bioinsecticide, viruses can control insect pests, such as baculoviruses [91]. Using these viruses as bioinsecticides has several advantages, including low cost, practicality, environmental friendliness, potency against agricultural pests, safety for animals due to their inability to multiply within animal tissue despite their ability to penetrate it, and a wide variety of hosts [92]. In a practical study, 40 different viruses were used as bioinsecticides. The results indicated that these viruses were more effective than chemical insecticides at controlling insect pests. Baculoviruses control insect pests by penetrating insect cells, multiplying and replicating rapidly, and causing cytotoxicity in their hosts [82].

## Conclusion

After reviewing this review article, we have come to the realization that the use of bacteriophages in agriculture to control bacterial plant diseases faces numerous challenges that may impede their successful application. However, we have also discussed potential solutions to overcome these obstacles. It is crucial to understand these problems and their resolutions before the implementation of bacteriophages in the agricultural sector to ensure their success.

In the future, we should explore the potential of viruses and their applications in various fields. Additionally, we must focus our attention on using these tools to combat global crises, such as the worldwide spread of antibiotic resistance.

## Data Availability

Not applicable.

## Abbreviations

CPMV: Cowpea mosaic virus
TMV: Tobacco mosaic virus
PVX: Potato virus
RM: Restriction/ Modification systems
BREX: Bacteriophage Exclusion system
DISARM: Defense island system associated with restriction-modification

## References

[1] Plattner F, Soldati-Favre D (2008). Hijacking of host cellular functions by the Apicomplexa. Annu Rev Microbiol.

[2] Brown N, Bhella D (2016). Are viruses alive. Microbiol Today.

[3] Adams MH. Bacteriophages. Interscience publishers; 1959. 10.5962/bhl.title.6966

[4] Clokie MR, Millard AD, Letarov AV, Heaphy S (2011). Phages in nature. Bacteriophage.

[5] Sakib, Sadman, and Samiha Kamal Shounak. The combination of bacteriophage therapy and antibiotic therapy. Diss. Brac University, 2021.

[6] Kassa T (2021). Bacteriophages against pathogenic bacteria and possibilities for future application in Africa. Infect Drug Resist.

[7] Guttman B, Raya R, Kutter E. Basic phage biology. Bacteriophages: Biology and applications. 2005;4:30–63. 10.1201/9780203491751.ch3

[8] Abuladze, Tamar, et al. "Bacteriophages reduce experimental contamination of hard surfaces, tomato, spinach, broccoli, and ground beef by Escherichia coli O157: H7." Applied and environmental microbiology 74.20 (2008): 6230–6238. 10.1128/AEM.01465-08 10.1128/AEM.01465-08 PMC2570303 18723643

[9] Lenski RE. Dynamics of interactions between bacteria and virulent bacteriophage. InAdvances in microbial ecology 1988 (pp. 1–44). Springer, Boston, MA. 10.1007/978-1-4684-5409-3_1

[10] Braun V, Hantke K. Bacterial receptors for phages and colicins as constituents of specific transport systems. InMicrobial interactions 1977 (pp. 99–137). Springer, Boston, MA. 10.1007/978-1-4615-9698-1_3

[11] Goldberg E. Bacteriophage nucleic acid penetration. InVirus receptors 1980 (pp. 115–141). Springer, Dordrecht. 10.1007/978-94-011-6918-9_6

[12] Xu J, Xiang Y (2017). Membrane penetration by bacterial viruses. J Virol.

[13] Warren RJ, Bose SK. Bacteriophage-Induced Inhibition of Host Functions: I. Degradation of Escherichia coli Deoxyribonucleic Acid after T4 Infection. J Virology. 1968;2(4):327–34. 10.1128/jvi.2.4.327-334.1968 10.1128/jvi.2.4.327-334.1968 PMC375618 4911847

[14] Young RY (1992). Bacteriophage lysis: mechanism and regulation. Microbiol Rev.

[15] Grabowski Ł, Łepek K, Stasiłojć M, Kosznik-Kwaśnicka K, Zdrojewska K, Maciąg-Dorszyńska M, Węgrzyn G, Węgrzyn A (2021). Bacteriophage-encoded enzymes destroying bacterial cell membranes and walls, and their potential use as antimicrobial agents. Microbiol Res.

[16] Davies EV, Winstanley C, Fothergill JL, James CE. The role of temperate bacteriophages in bacterial infection. FEMS microbiology letters. 2016 Mar 1;363(5). 10.1093/femsle/fnw015 10.1093/femsle/fnw015 26825679

[17] Zabriskie JB (1964). The role of temperate bacteriophage in the production of erythrogenic toxin by group A streptococci. J Exp Med.

[18] Kuhl S, Hyman P, Abedon ST. Diseases caused by phages. Bacteriophages in health and disease. 2012:21–32. 10.1079/9781845939847.0021

[19] Brüssow Harald, Canchaya C, Hardt W-D. Phages and the evolution of bacterial pathogens: From genomic rearrangements to Lysogenic conversion. Microbiology and Molecular Biology Reviews. 2004;68(3):560–602. 10.1128/MMBR.68.3.560-602.2004 10.1128/MMBR.68.3.560-602.2004 PMC515249 15353570

[20] NIAID’s Antibacterial Resistance Program: Current Status and Future Directions. https://www.niaid.nih.gov/sites/default/files/arstrategicplan2014.pdf: National Institutes of Health; 2014

[21] Loc-Carrillo C, Abedon ST (2011). Pros and cons of phage therapy. Bacteriophage.

[22] Weber-Dąbrowska B, Jończyk-Matysiak E, Żaczek M, Łobocka M, Łusiak-Szelachowska M, Górski A (2016). Bacteriophage procurement for therapeutic purposes. Front Microbiol.

[23] Jones, Jeffrey B., et al. "Bacteriophages for plant disease control." Annu. Rev. Phytopathol. 45 (2007): 245–262. 10.1146/annurev.phyto.45.062806.094411 10.1146/annurev.phyto.45.062806.094411 17386003

[24] Buttimer C, McAuliffe O, Ross RP, Hill C, O’Mahony J, Coffey A (2017). Bacteriophages and bacterial plant diseases. Front Microbiol.

[25] Lohr L. Factors affecting international demand and trade in organic food products. Changing structure of global food consumption and trade. 2001:67–79

[26] Iriarte, F. B., et al. "Factors affecting survival of bacteriophage on tomato leaf surfaces." *Applied and environmental microbiology* 73.6 (2007): 1704–1711.11. 10.1128/AEM.02118-06 10.1128/AEM.02118-06 PMC1828813 17259361

[27] Born, Yannick, et al. "Protection of Erwinia amylovora bacteriophage Y2 from UV-induced damage by natural compounds." *Bacteriophage* 5.4 (2015): e1074330. 10.1080/21597081.2015.1074330 10.1080/21597081.2015.1074330 PMC4743488 26904378

[28] Khalil, Ibrahim R., et al. "Poly-γ-glutamic acid: biodegradable polymer for potential protection of beneficial viruses." *Materials* 9.1 (2016): 28. 10.3390/ma9010028 10.3390/ma9010028 PMC5456517 28787828

[29] Wdowiak, Mateusz, et al. "Congo red protects bacteriophages against UV irradiation and allows for the simultaneous use of phages and UV for membrane sterilization." *Environmental Science: Water Research & Technology* 9.3 (2023): 696–706

[30] Templeton MR, Andrews RC, Hofmann R (2006). Impact of iron particles in groundwater on the UV inactivation of bacteriophages MS2 and T4. J Appl Microbiol.

[31] Templeton MR, Andrews RC, Hofmann R (2005). Inactivation of particle-associated viral surrogates by ultraviolet light. Water Res.

[32] Prashar P, Kapoor N, Sachdeva S (2014). Rhizosphere: its structure, bacterial diversity and significance. Reviews Environmental Sci Bio/Technology.

[33] Gupta, Sonal, and Ashwini A. Waoo. "Isolation and characterization of bacteria possessing Osmotolerance activity from phylloplane of Centella asiatica." *Journal of Applied Biology and Biotechnology* (2023). 10.1016/j.cub.2020.07.037

[34] Jones JB, Svircev AM, Obradović AŽ. Crop use of bacteriophages. Bacteriophages: biology, technology, therapy. 2021:839–56. 10.1007/978-3-319-41986-2_28

[35] Balogh B, Jones JB, Iriarte FB, Momol MT (2010). Phage therapy for plant disease control. Curr Pharm Biotechnol.

[36] Nagai, Hirofumi, et al. "Improved control of black rot of broccoli caused by Xanthomonas campestris pv. campestris using a bacteriophage and a nonpathogenic Xanthomonas sp. strain." Journal of General Plant Pathology 83 (2017): 373–381

[37] Tanaka H, Negishi H, Maeda H (1990). Control of tobacco bacterial wilt by an avirulent strain of Pseudomonas solanacearum M4S and its bacteriophage. Jpn J Phytopathol.

[38] Balogh B (2003). Improved efficacy of newly formulated bacteriophages for management of bacterial spot on tomato. Plant Dis.

[39] Svircev A, Roach D, Castle A (2018). Framing the future with bacteriophages in agriculture. Viruses.

[40] Yamada, Takashi. "Bacteriophages of Ralstonia solanacearum: their diversity and utilization as biocontrol agents in agriculture." Bacteriophages (2012): 113–138

[41] Álvarez B, López MM, Biosca EG (2019). Biocontrol of the major plant pathogen Ralstonia solanacearum in irrigation water and host plants by novel waterborne lytic bacteriophages. Front Microbiol.

[42] Thapa Magar, Roniya, et al. "Biocontrol of bacterial wilt in tomato with a cocktail of lytic bacteriophages." Applied microbiology and biotechnology 106.9–10 (2022): 3837–3848 10.1007/s00253-022-11962-7 35562488

[43] Kotay, Shireen M., et al. "Biocontrol of biomass bulking caused by Haliscomenobacter hydrossis using a newly isolated lytic bacteriophage." Water research 45.2 (2011): 694–704 10.1016/j.watres.2010.08.038 20950835

[44] Bae, Ju Young, et al. "Biocontrol potential of a lytic bacteriophage PE204 against bacterial wilt of tomato." J. Microbiol. Biotechnol 22.12 (2012): 1613–1620 10.4014/jmb.1208.08072 23221522

[45] Czajkowski, Robert, Anna Smolarska, and Zofia Ozymko. "The viability of lytic bacteriophage ΦD5 in potato-associated environments and its effect on Dickeya solani in potato (Solanum tuberosum L.) plants." PLoS One 12.8 (2017): e0183200 10.1371/journal.pone.0183200 PMC5553641 28800363

[46] Elhalag, Kamel, et al. "Potential use of soilborne lytic Podoviridae phage as a biocontrol agent against Ralstonia solanacearum." Journal of basic microbiology 58.8 (2018): 658–669 10.1002/jobm.201800039 29938804

[47] Ramírez M, Neuman BW, Ramírez CA (2020). Bacteriophages as promising agents for the biological control of moko disease (Ralstonia solanacearum) of banana. Biol Control.

[48] Korniienko, Nataliia, et al. "Phages of phytopathogenic bacteria: High potential, but challenging application." Plant Protection Science 58.2 (2022): 81–91

[49] Ross A, Ward S, Hyman P (2016). More is better: selecting for broad host range bacteriophages. Front Microbiol.

[50] Baliyan, Nitin, et al. "Bacteriophage cocktails as antibacterial agents in crop protection." Environmental Sustainability 5.3 (2022): 305–311

[51] Iriarte, Fanny B., et al. "Soil-based systemic delivery and phyllosphere in vivo propagation of bacteriophages: two possible strategies for improving bacteriophage persistence for plant disease control." Bacteriophage 2.4 (2012): e23530 10.4161/bact.23530 PMC3594209 23532156

[52] Wang, Xiaofang, et al. "Phage combination therapies for bacterial wilt disease in tomato." Nature Biotechnology 37.12 (2019): 1513–1520 10.1038/s41587-019-0328-3 31792408

[53] Wei, Cuihua, et al. "Developing a bacteriophage cocktail for biocontrol of potato bacterial wilt." Virologica sinica 32 (2017): 476–484 10.1007/s12250-017-3987-6 PMC6598922 29168148

[54] Liu, Na, et al. "Phage cocktail therapy: multiple ways to suppress pathogenicity." Trends in plant science 25.4 (2020): 315–317 10.1016/j.tplants.2020.01.013 32191865

[55] Farooq, Tahir, et al. "Deploying viruses against phytobacteria: Potential use of phage cocktails as a multifaceted approach to combat resistant bacterial plant pathogens." Viruses 14.2 (2022): 171 10.3390/v14020171 PMC8879233 35215763

[56] Zaczek-Moczydłowska, Maja A., et al. "Phage cocktail containing Podoviridae and Myoviridae bacteriophages inhibits the growth of Pectobacterium spp. under in vitro and in vivo conditions." PLoS One 15.4 (2020): e0230842 10.1371/journal.pone.0230842 PMC7117878 32240203

[57] Carstens, Alexander Byth, et al. "A novel six-phage cocktail reduces Pectobacterium atrosepticum soft rot infection in potato tubers under simulated storage conditions." FEMS Microbiology Letters 366.9 (2019): fnz101 10.1093/femsle/fnz101 31095303

[58] Bugaeva, Eugenia N., et al. "Use of a specific phage cocktail for soft rot control on ware potatoes: A case study." Viruses 13.6 (2021): 1095 10.3390/v13061095 PMC8229397 34201375

[59] Geary N (2013). Understanding synergy. Am J Physiol Endocrinol Metab.

[60] Schmerer M, Molineux IJ, Bull JJ (2014). Synergy as a rationale for phage therapy using phage cocktails. PeerJ.

[61] Kering, Kelvin K., et al. "Application of adaptive evolution to improve the stability of bacteriophages during storage." *Viruses* 12.4 (2020): 423. 10.3390/v12040423 10.3390/v12040423 PMC7232334 32283683

[62] Jończyk-Matysiak, Ewa, et al. "Factors determining phage stability/activity: Challenges in practical phage application." *Expert Review of Anti-infective Therapy* 17.8 (2019): 583–606. 10.1080/14787210.2019.1646126 10.1080/14787210.2019.1646126 31322022

[63] Zhang, Yajie, et al. "Manufacturing and ambient stability of shelf freeze dried bacteriophage powder formulations." International journal of pharmaceutics 542.1–2 (2018): 1–7 10.1016/j.ijpharm.2018.02.023 29486286

[64] Leung, Sharon SY, et al. "Effect of storage temperature on the stability of spray dried bacteriophage powders." European Journal of Pharmaceutics and Biopharmaceutics 127 (2018): 213–222 10.1016/j.ejpb.2018.02.033 PMC5948144 29486303

[65] Chang, Rachel Yoon Kyung, et al. "Storage stability of inhalable phage powders containing lactose at ambient conditions." International journal of pharmaceutics 560 (2019): 11–18 10.1016/j.ijpharm.2019.01.050 PMC6502640 30710661

[66] Mojica, Francisco JM, et al. "Intervening sequences of regularly spaced prokaryotic repeats derive from foreign genetic elements." *Journal of molecular evolution* 60 (2005): 174–182. 10.1007/s00239-004-0046-3 10.1007/s00239-004-0046-3 15791728

[67] Scanlan, Pauline D., et al. "Coevolution with bacteriophages drives genome-wide host evolution and constrains the acquisition of abiotic-beneficial mutations." *Molecular biology and evolution* 32.6 (2015): 1425–1435 10.1093/molbev/msv032 10.1093/molbev/msv032 PMC6281048 25681383

[68] Ofir, Gal, et al. "DISARM is a widespread bacterial defence system with broad anti-phage activities." *Nature microbiology* 3.1 (2018): 90–98. 10.1038/s41564-017-0051-0 10.1038/s41564-017-0051-0 PMC5739279 29085076

[69] Frampton, Rebekah A., et al. "Identification of bacteriophages for biocontrol of the kiwifruit canker phytopathogen Pseudomonas syringae pv. actinidiae." *Applied and Environmental Microbiology* 80.7 (2014): 2216–2228. 10.1128/AEM.00062-14 10.1128/AEM.00062-14 PMC3993152 24487530

[70] Roach, Dwayne R., et al. "Absence of lysogeny in wild populations of E rwinia amylovora and P antoea agglomerans." *Microbial biotechnology* 8.3 (2015): 510–518. 10.1111/1751-7915.12253 10.1111/1751-7915.12253 PMC4408183 25678125

[71] Goldfarb, Tamara, et al. "BREX is a novel phage resistance system widespread in microbial genomes." *The EMBO journal* 34.2 (2015): 169–183. 10.15252/embj.201489455 10.15252/embj.201489455 PMC4337064 25452498

[72] Tock, Mark R., and David TF Dryden. "The biology of restriction and anti-restriction." *Current opinion in microbiology* 8.4 (2005): 466–472. 10.1016/j.mib.2005.06.003 10.1016/j.mib.2005.06.003 15979932

[73] Doron, Shany, et al. "Systematic discovery of antiphage defense systems in the microbial pangenome." *Science* 359.6379 (2018): eaar4120. 10.1126/science.aar4120 10.1126/science.aar4120 PMC6387622 29371424

[74] Kering KK, Kibii BJ, Wei H (2019). Biocontrol of phytobacteria with bacteriophage cocktails. Pest Manag Sci.

[75] Sabri, Miloud, et al. "Phages as a potential biocontrol of phytobacteria." Archives of Phytopathology and Plant Protection 54.17–18 (2021): 1277–1291

[76] Kim, Hyeongsoon, et al. "Development of a bacteriophage cocktail against Pectobacterium carotovorum subsp. carotovorum and its effects on Pectobacterium virulence." Applied and Environmental Microbiology 88.19 (2022): e00761–22 10.1128/aem.00761-22 PMC9552609 36165651

[77] Labrie SJ, Samson JE, Moineau S (2010). Bacteriophage resistance mechanisms. Nat Rev Microbiol.

[78] Kilcher S, Loessner MJ (2019). Engineering bacteriophages as versatile biologics. Trends Microbiol.

[79] Sieiro, Carmen, et al. "A hundred years of bacteriophages: Can phages replace antibiotics in agriculture and aquaculture?." Antibiotics 9.8 (2020): 493 10.3390/antibiotics9080493 PMC7460141 32784768

[80] Jones, Jeffrey B., et al. "Considerations for using bacteriophages for plant disease control." *Bacteriophage* 2.4 (2012): e23857. 10.4161/bact.23857 10.4161/bact.23857 PMC3594208 23531902

[81] Grace, Emily R., et al. "Seeing the forest for the trees: Use of phages to treat bacterial tree diseases." *Plant Pathology* 70.9 (2021): 1987–2004. 10.1111/ppa.13465

[82] Gizaw, Birhanu. "Corona and Other Virus: Their Useful and Harmful Aspects." *Journal of Virology Research & Reports. SRC/JVRR-103* (2020): 3. 10.47363/JVRR/2020(1)105

[83] Thangavelu, Raja Muthuramalingam, et al. "Fabrication of virus metal hybrid nanomaterials: An ideal reference for bio semiconductor." *Arabian Journal of Chemistry* 13.1 (2020): 2750–2765. 10.1016/j.arabjc.2018.07.006

[84] Lico C (2013). Nanoparticles in biomedicine: new insights from plant viruses. Curr Med Chem.

[85] Daniell, Henry, et al. "Plant-made vaccine antigens and biopharmaceuticals." *Trends in plant science* 14.12 (2009): 669–679. 10.1016/j.tplants.2009.09.009 10.1016/j.tplants.2009.09.009 PMC2787751 19836291

[86] Tatsis, Nia, and Hildegund CJ Ertl. "Adenoviruses as vaccine vectors." *Molecular Therapy* 10.4 (2004): 616–629. 10.1016/j.ymthe.2004.07.013 10.1016/j.ymthe.2004.07.013 PMC7106330 15451446

[87] Hartman ZC, Appledorn DM, Amalfitano A (2008). Adenovirus vector induced innate immune responses: impact upon efficacy and toxicity in gene therapy and vaccine applications. Virus Res.

[88] Lundstrom K (2001). Alphavirus vectors for gene therapy applications. Curr Gene Ther.

[89] Lundstrom K (2005). Biology and application of alphaviruses in gene therapy. Gene Ther.

[90] Levine, Beth, et al. "Conversion of lytic to persistent alphavirus infection by the bcl-2 cellular oncogene." *Nature* 361.6414 (1993): 739–742. 10.1038/361739a0. 10.1038/361739a0 8441470

[91] Bonning, Bryony C., and Tyasning Nusawardani. "Introduction to the use of baculoviruses as biological insecticides." *Baculovirus and Insect Cell Expression Protocols* (2007): 359–366. 10.1007/978-1-59745-457-5_18 10.1007/978-1-59745-457-5_18 17951780

[92] Beas-Catena, A., et al. "Baculovirus biopesticides: an overview." *JAPS: Journal of Animal & Plant Sciences* 24.2 (2014)

